# Exploring natural killer cell-related biomarkers in multiple myeloma: a novel nature killer cell-related model predicting prognosis and immunotherapy response using single-cell study

**DOI:** 10.1007/s10238-024-01322-2

**Published:** 2024-04-18

**Authors:** Jing Zhao, Xiaoning Wang, Huachao Zhu, Suhua Wei, Hailing Zhang, Le Ma, Wenjuan Zhu

**Affiliations:** 1 Department of Hematology, The First Affiliated Hospital of Xi’an Jiaotong University, 277 Yanta West Road, Xi’an, 710061 Shaanxi People’s Republic of China; 2 Department of Medical, Xi’an Gem Flower Changqing Hospital, No. 20 Changqing West Road, Xi’an, 710201 Shaanxi People’s Republic of China

**Keywords:** Multiple myeloma, Natural killer cells, Single-cell sequencing, Bulk transcription, Immune microenvironment

## Abstract

**Background:**

Natural killer cells (NKs) may be involved in multiple myeloma (MM) progression. The present study elucidated the correlation between NKs and the progression of MM using single-cell binding transcriptome probes to identify NK cell-related biomarkers.

**Methods:**

Single-cell analysis was performed including cell and subtype annotation, cell communication, and pseudotime analysis. Hallmark pathway enrichment analysis of NKs and NKs-related differentially expressed genes (DEGs) were conducted using Gene Ontology, Kyoto Encyclopedia of Genes and Genomes, and protein–protein interaction (PPI) networks. Then, a risk model was structured based on biomarkers identified through univariate Cox regression analysis and least absolute shrinkage and selection operator regression analysis and subsequently validated. Additionally, correlation of clinical characteristics, gene set enrichment analysis, immune analysis, regulatory network, and drug forecasting were explored.

**Results:**

A total of 13 cell clusters were obtained and annotated, including 8 cell populations that consisted of NKs. Utilizing 123 PPI network node genes, 8 NK-related DEGs were selected to construct a prognostic model. Immune cell infiltration results suggested that 11 immune cells exhibited marked differences in the high and low-risk groups. Finally, the model was used to screen potential drug targets to enhance immunotherapy efficacy.

**Conclusion:**

A new prognostic model for MM associated with NKs was constructed and validated. This model provides a fresh perspective for predicting patient outcomes, immunotherapeutic response, and candidate drugs.

**Supplementary Information:**

The online version contains supplementary material available at 10.1007/s10238-024-01322-2.

## Introduction

Multiple myeloma (MM) is an incurable hematological malignancy that is characterized by the uncontrolled clonal proliferation of malignant plasma cells in the bone marrow [1]. The worldwide incidence of MM in 2020 was 1.78 per 100,000 individuals, with a mortality rate of 1.14 per 100,000 individuals [2]. The overall survival for MM patients increased from 32% in 1996 to 54% in 2020 due to the wide application of new and targeted drugs, including proteasome inhibitors, immunomodulators, monoclonal antibodies, and other immune therapeutic regimens [3]. However, patients ultimately experience disease progression despite initial responses. Notably, the interaction between malignant plasma cells and the highly immunosuppressive tumor microenvironment (TME) of the bone marrow is a significant determinant of tumorigenesis and the occurrence of chemoresistance [4]. Natural killer cells play a crucial role in the immune surveillance of tumors due to their inherent ability to induce tumor cell lysis. Studies in mouse models and clinical subjects demonstrated a correlation between reduced NK activity and increased susceptibility to cancer and metastasis [5–7]. NK cells release cytotoxic granules, which activate death receptor-mediated pathways, such as FasL/Fas, to trigger target cell death [8]. Meanwhile, NK cells secrete chemokines and cytokines that exert immunomodulatory functions [9]. The single-cell transcriptome has become an indispensable approach to elucidating the intricate landscape of biological heterogeneous tumor cells and the immune microenvironment in MM [10, 11]. According to a single-cell RNA sequencing study, NK cell populations are enriched during MGUS but depleted in the later stages of MM. This changed phenotype suggests an impaired immune system [12]. Differences have also been observed in NK cell function, with reduced or elevated NK cell function being associated with advanced clinical stages of MM, high-risk disease, and survival [13, 14]. Although an effect of NK cells on the outcome of MM was demonstrated, there is limited information on the prognostic value of NK cell-related genes in MM. The present paper presents a series of analyses that identified biomarkers associated with NK cell function in the progression of MM using single-cell sequencing and bulk transcription approaches. Based on these analyses, a novel prognostic model for MM associated with NK cells was constructed and validated. The results offer a fresh perspective for investigating the relationship between NK cells and MM and identifying potential targets for immune therapeutic strategies.

## Materials and methods

### Data source

Three multiple myeloma (MM) datasets, GSE176131 (GPL24676), GSE136324 (GPL27143), and GSE136337 (GPL27143), were mined from the Gene Expression Omnibus (GEO) database. GSE176131 was a single-cell dataset containing 9 MM samples and 2 control samples. The training set GSE136324 contained 866 cancer samples with survival information. The external validation set GSE136337 had 424 cancer samples with survival information.

### Single-cell sequencing data analysis

Data from the single-cell dataset were filtered using Seurat (v 4.0.5) [15]. The cell filtering conditions were library size and gene count of 2 times the median absolute deviation (MAD) and mitochondrial content > 20%. The highly variable genes within cells after log-normalization were retrieved for subsequent analysis using the VST method of the FindVariableFeatures function. A subset of principal components obtained from the principal component analysis (PCA) of the integrated sample was selected for subsequent analysis. The unsupervised clustering analysis of cells was performed using the FindNeighbors and FindClusters functions within the Seurat package. The results of the clustering were visualized using t-distributed stochastic neighbor embedding (t-SNE) and uniform manifold approximation and projection (UMAP). To identify significant markers, the FindAllMarkers function was used with a minimum percentage threshold of 0.5 and a logarithmic fold change threshold of 0.5 to determine cell subcluster types. The marker genes of each cell cluster were compared to the marker genes of each cell type in the CellMarker database (http://xteam.xbio.top/CellMarker/) [16]. Cell types were annotated using SingleR (v 1.0.6) [17]. Differential cell clusters containing NK cells from MM and control samples were further screened for subsequent analysis.

### Hallmark pathway enrichment and cell communication analysis

To assess the functional differences of NKs between normal and MM samples, Hallmark pathway enrichment analysis of NKs was performed using GSVA (v 1.46.0) [18]. The differential Hallmark pathways inter-cell clusters were screened using limma (v 3.42.2) [18] with -log_10_*P* > 1.3, and visualization was performed using ggplot2 (v 3.3.6) [19] and heatmap (v 1.0.12) [20]. Communication and interactions between cells were analyzed in CellChat (v 1.6.1) [21], and detailed networks of each cellular interaction are shown using shell diagrams.

### *Differentially expressed gene* (*DEG)-NK screening and functional analysis*

The NK data were extracted from GSE176131, and the FindMarkers function was used to identify DEGs in NKs between MM samples and healthy control samples (|avg_log_2_FC|> 0.5). The DEGs underwent GO analysis, including biological process (BP), molecular functions (MF), and cellular components (CC), followed by Kyoto Encyclopedia of Genes and Genomes (KEGG) enrichment analysis, which was performed using DAVID (v 4.4.4) [22]. A PPI network was constructed using the DEGs and the STRING website (https://cn.string-db.org/). Based on the DEGs, the STRING website was used to construct a PPI network.

### Construction and validation of risk model

Biomarkers in GSE136324 were selected using univariate Cox regression and least absolute shrinkage and selection operator (LASSO) regression analysis with glmnet (v 4.0–2) [23] based on PPI network node genes. A risk model was constructed in accordance with biomarker expression and overall survival (OS), and risk scores were assessed using the following formula.

$${\text{Risk}} {\text{score}}=\sum_{n=1}^{n}({\text{coefi}}*Xi)$$

Coef and X denote coefficients and gene expression, respectively. The GSE136324 samples were sorted into high- and low-risk groups by risk score median. Kaplan–Meier (K-M) survival curves in both risk groups were drawn using survival (v 3.2–7) [24]. To ensure the validity of the risk model, 1-, 3-, and 5-year receiver operating characteristic (ROC) curves were created, and area under the curve (AUC) values were computed using survivalROC (v 1.0.3) [25]. GSE136337 was the external validation set for model validation.

### Correlation analysis of risk model and clinical characteristics and independent prognostic analysis

The significance of differentiation between risk scores and clinical characteristics was analyzed. Survival analyses of different stratified clinical characteristics were performed in the two risk groups. Sex, age, albumin, b2m, LDH, ISS, and risk score were included in the univariate Cox, PH test, and multivariate Cox regression analysis. RMS (v 6.4–1) [26] was used to construct a nomogram of survival at 1, 3, and 5 years for the clinical factors in the multivariate Cox model described above. The predictive ability of the nomogram was calculated from the calibration and ROC curves.

### Correlation of biomarkers and immune cell infiltration analysis

GSEA (v 4.0.3) [27] was used to acquire biomarkers related to dramatically enriched pathways based on the Hallmark background gene set. The single-sample GSEA (ssGSEA) algorithm was used to assess the abundance of immune cell infiltration in all samples from the two risk groups. The correlation between the differential immune cells was computed using the Spearman method. Differences in immune checkpoints (ICs), MHC molecules, chemokines, and receptor-associated genes in both risk groups were analyzed using the ggplot2 package (v 3.3.6) [19].

### NKs differentiation trajectories, regulatory networks, chemo drug prediction, and survival analysis

The differentialGeneTest function was performed to identify NK genes that differed between MM and control samples, and the proposed pseudotime analysis was performed using Monocle (v 2.18.0) [28]. MiRNA and transcription factor (TF) prediction analyses of biomarkers were performed using the NetworkAnalyst database (https://www.networkanalyst.ca/), Gene-miRNA interaction database: miRTarBase v8.0. MiRNA-mRNA-TF regulatory network was created using Cytoscape (v 3.6.1) [29]. Correlation analysis of biomarkers and TFs was performed using the Spearman method. The 50% inhibitory concentration (IC_50_) values of 138 cancer cell line drugs with specific names were compared according to Genomics of Drug Sensitivity in Cancer (GDSC, https://www.cancerrxgene.org/) using pRRophetic (v 0.5) [30]. MM samples in GSE136324 were divided into high and low-expression cohorts based on the median level of each biomarker expression, and K-M survival analysis was performed using the survival package (v 3.2–7) [31].

### Patients and samples

Patients were diagnosed using the criteria established by the International Myeloma Working Group for MM. The clinical stage and risk status of MM patients were determined according to the Revised International Staging System. Healthy donors were included as controls after obtaining informed consent. Bone marrow samples were collected from patients and healthy controls, and the mononuclear cells were separated using lymphocyte separation liquid.

### Quantitative real-time polymerase chain reaction (qRT-PCR)

Total cellular RNA was extracted using TRIzol reagent (Invitrogen, CA, USA). The extracted total RNA was reverse transcribed to cDNA using HiScript® II Q RT SuperMix for qPCR (Vazyme Biotech, Nanjing, China) with random primers for mRNA. RT-PCR analysis was performed using SYBR Green Master Mix reagents (Vazyme Biotech, Nanjing, China) to amplify and analyze the expression of mRNA. The sequences of primers used are provided in Supplement Table 1. The relative expression of genes was determined using the 2^−ΔΔCT^ method.

### Statistical analysis

Analyses of public data were performed using the R programming language, and differential analysis comparisons were performed using the Wilcox test. The Kruskal‒Wallis test was used for multiple group comparisons. All experiments were replicated three times, and the results are presented as the mean ± SEM using GraphPad Prism (9.3.0). Statistical significance was determined using a t test and one-way ANOVA. All results were considered statistically significant when *P* < 0.05.

## Results

### Identification of NKs at single-cell level

**Fig. 1:**
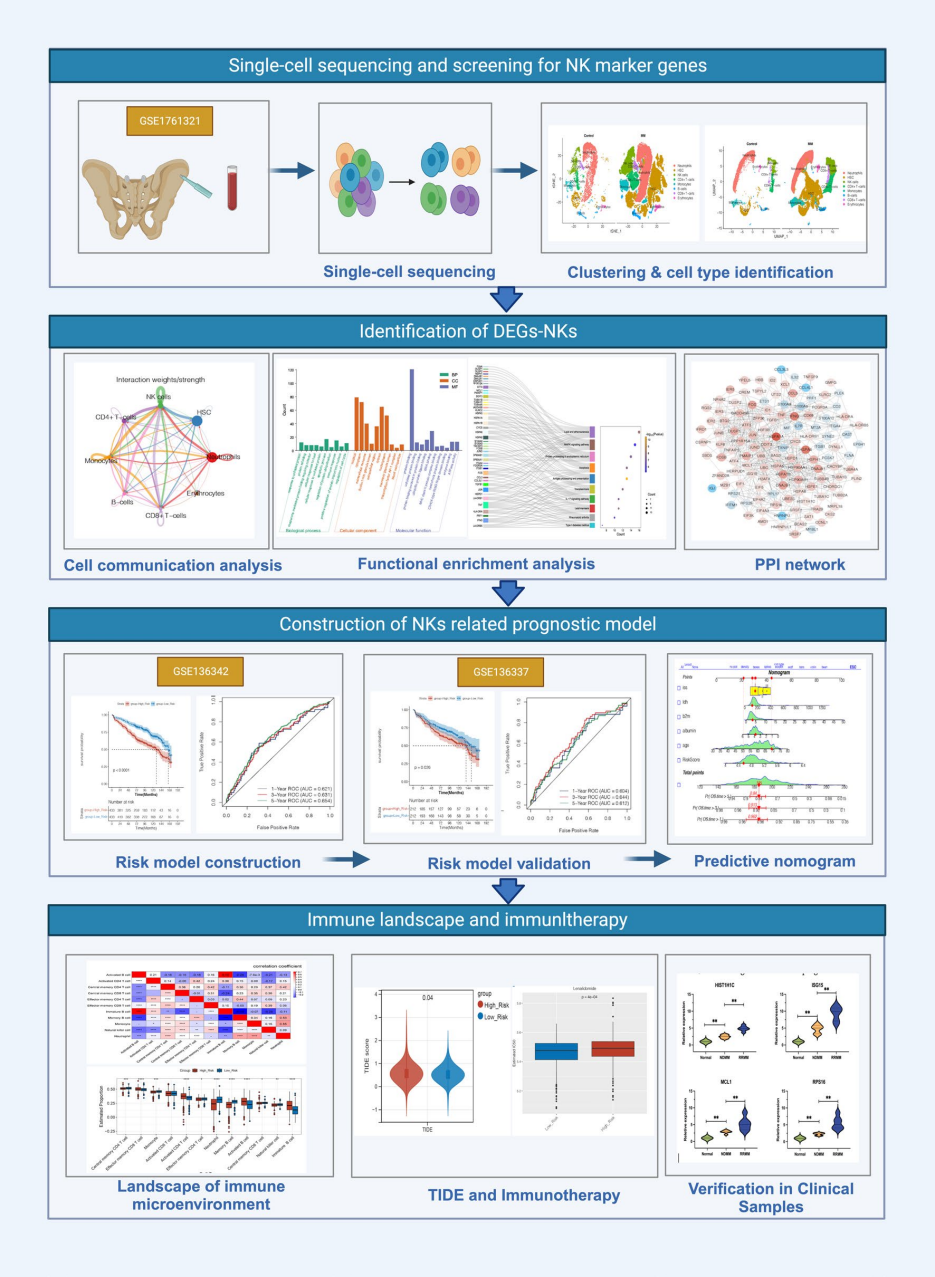
Flow chart of this study

**Fig. 2:**
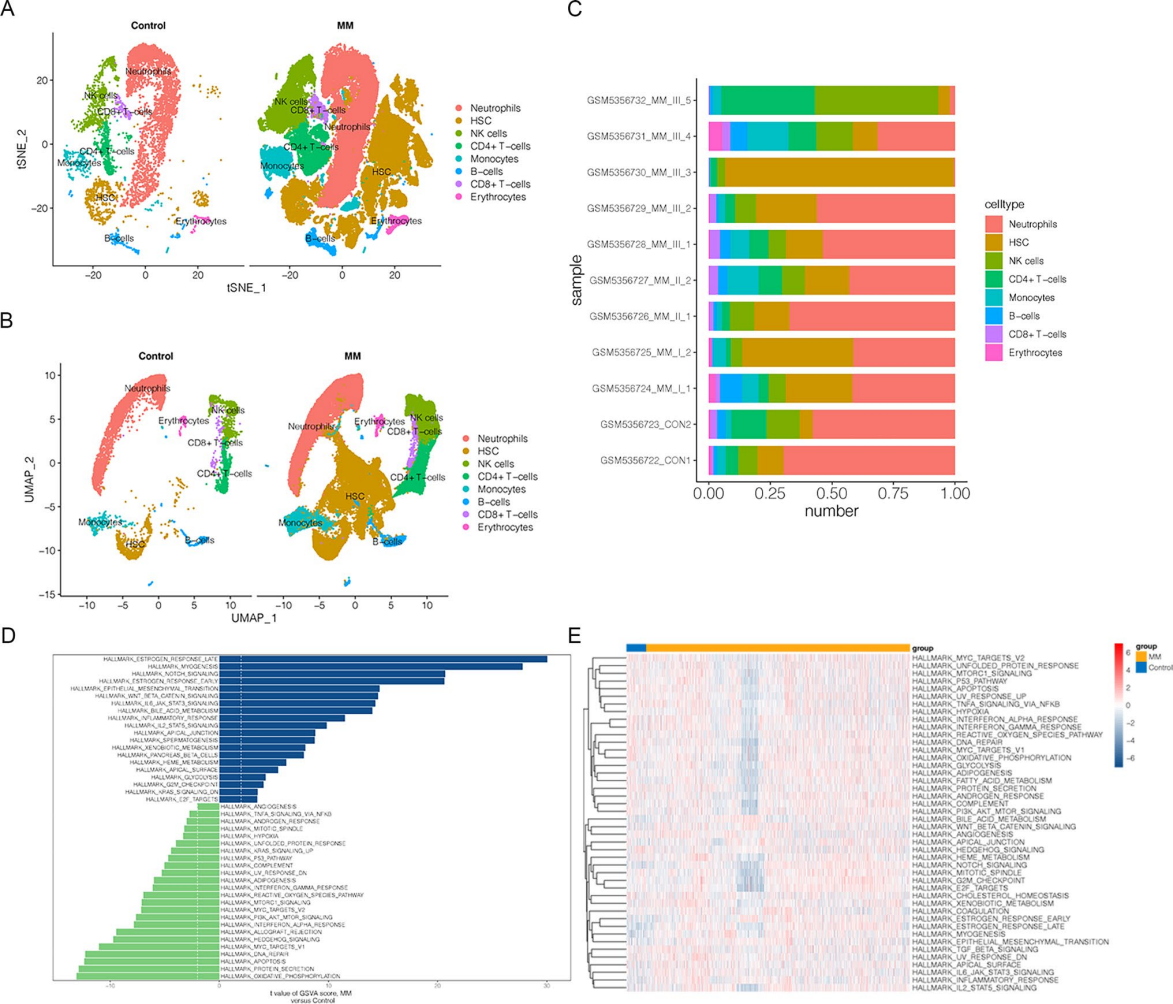
The identification of cell clusters based on the profile of a single-cell. **a** The T-SNE visualization of cluster distribution. **b** Visualization of cluster distribution using UMAP. **c** The bar diagrams represent the distribution of 8 types of cells in each sample. **d** Hallmark pathway of MM and control groups by GSVA enrichment analysis. **e** Heatmap visualization of Hallmark pathway enrichment

A brief flowchart depicting this study is displayed in Fig. 1. After quality control (QC) of single-cell GSE104782 data, a total of 11,517 cells and 2,000 highly variable genes were screened. Thirty principal components were selected by PCA, and t-SNE and UMAP plots showed no obvious batch in 13 clusters and each sample. Eight cell populations (neutrophils, NKs, HSCs, CD4 + T cells, monocytes, B cells, CD8 + T cells, and erythrocytes) were annotated from 13 cell clusters for subsequent analysis (Fig. 2a–b). The percentage of cell populations in each sample source was counted (Fig. 2c). Hallmark pathway enrichment analysis screened 44 pathways, including ‘IL6-JAK-STAT3 signaling’, ‘P53 pathway’, ‘PI3K AKT MTOR signaling’, ‘MYC TARGETS V1’, and other immune-related pathways that differed between NKs (Fig. 2d–e).

### Communication between NK cells and other immune cells

**Fig. 3:**
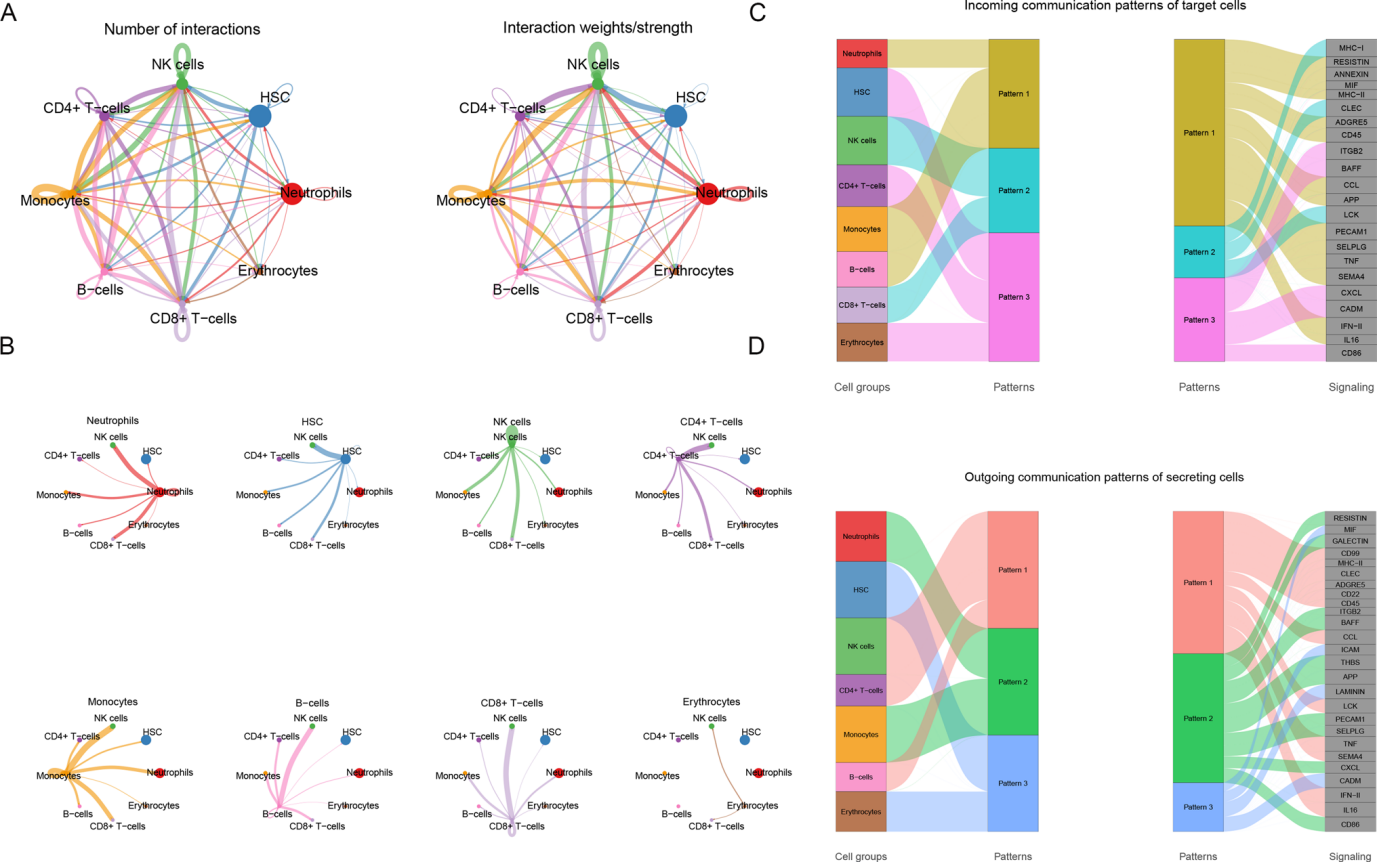
Communication between NK cells and other immune cells analyzed using cell chat. **a** Cell–cell interactions in 8 cell types. Direction is indicated by arrows, and edge thickness is a measure of cell interaction. **b** Shell diagram of cell–cell interactions maps including each cellular interaction. **c–d** Sankey Diagram of incoming and outgoing communication patterns between different cell subtypes and signaling pathways

Ligand-receptor network relationships for cellular communication were constructed to infer communication probabilities at the pathway level, and detailed networks for each cellular interaction are shown in shell diagrams. Strong communication between NKs and monocytes and CD8 + T cells was identified(Fig. 3a–b). Alluvial plots showed the association with cell subpopulations, ligand-receptor pairs, or signaling pathways for incoming and outgoing communication modes (Fig. 3c–d).

### Identification and functional analysis of DEGs-NKs

**Fig. 4:**
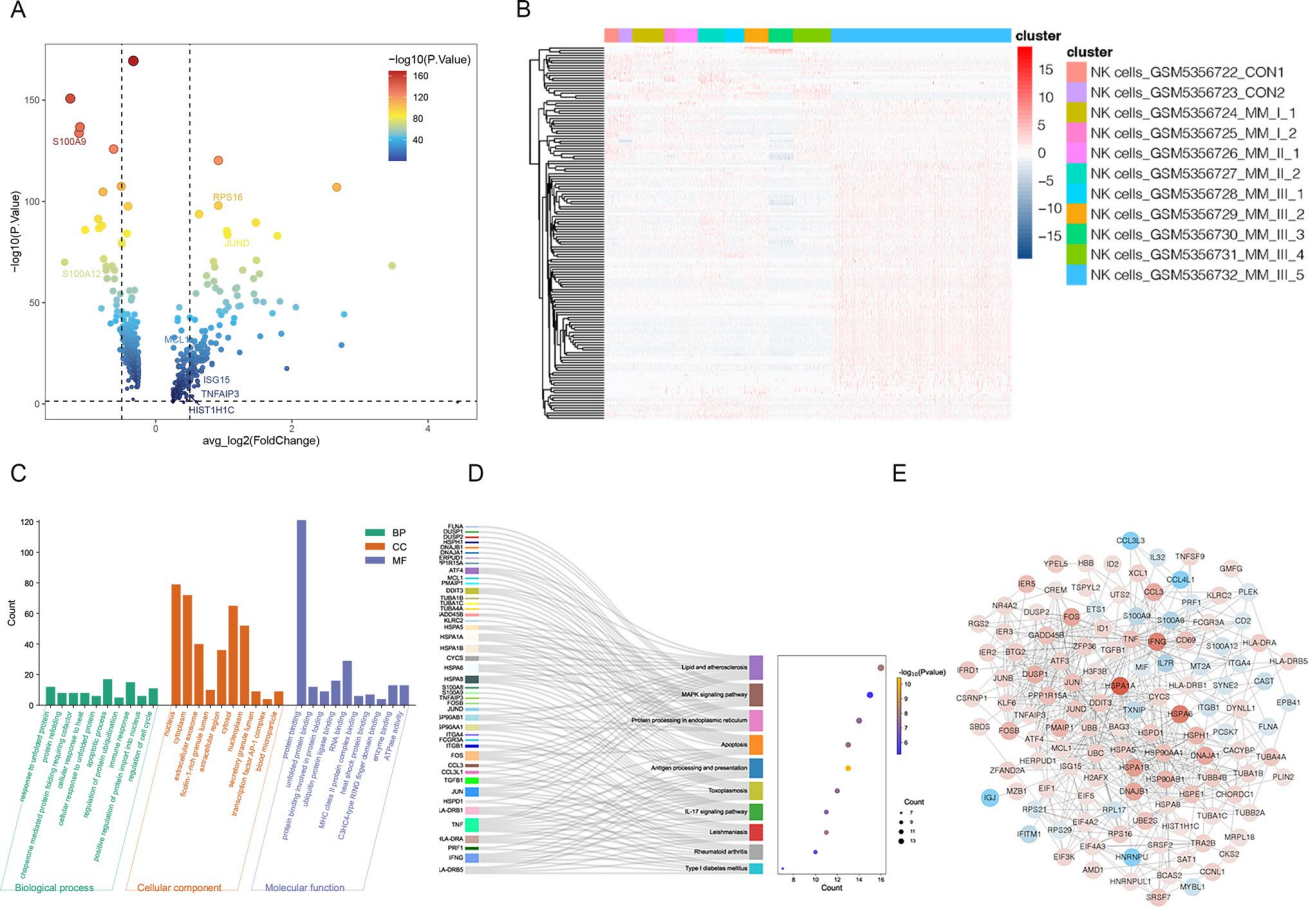
Identification and function analysis for DEGs-NKs. **a** A volcano plot of NK cell-associated genes that were differentially expressed between MM and Control samples. **b** Clustering heatmap of differential NK cell marker genes in MM and Control samples. **c** GO pathway enrichment analysis for DEGs-NKs. **d** The Sankey and Bubble plot of KEGG analysis including the top 10 KEGG enrichment pathways of DEGs-NKs. **e** Networking of DEGs-NKs based on protein–protein interactions (PPIs)

A total of 148 DEGs-NKs between MM and control samples were selected in GSE176131 (Fig. 4a–b). To examine the functions of DEGs-NKs, we analyzed the active pathways in each gene (Fig. 4c–d). According to the GO analysis, the DEGs-NKs were enriched in the apoptotic process, immune response, and regulation of the cell cycle. KEGG pathway analysis revealed that most KEGG components were involved in the IL-17 signaling pathway, apoptosis, and MAPK signaling pathway. The PPI network demonstrated 651 protein–protein pairs of 123 proteins (Fig. 4e).

### Potential candidate biomarkers related to DEGs-NKs were identified and validated

**Fig. 5:**
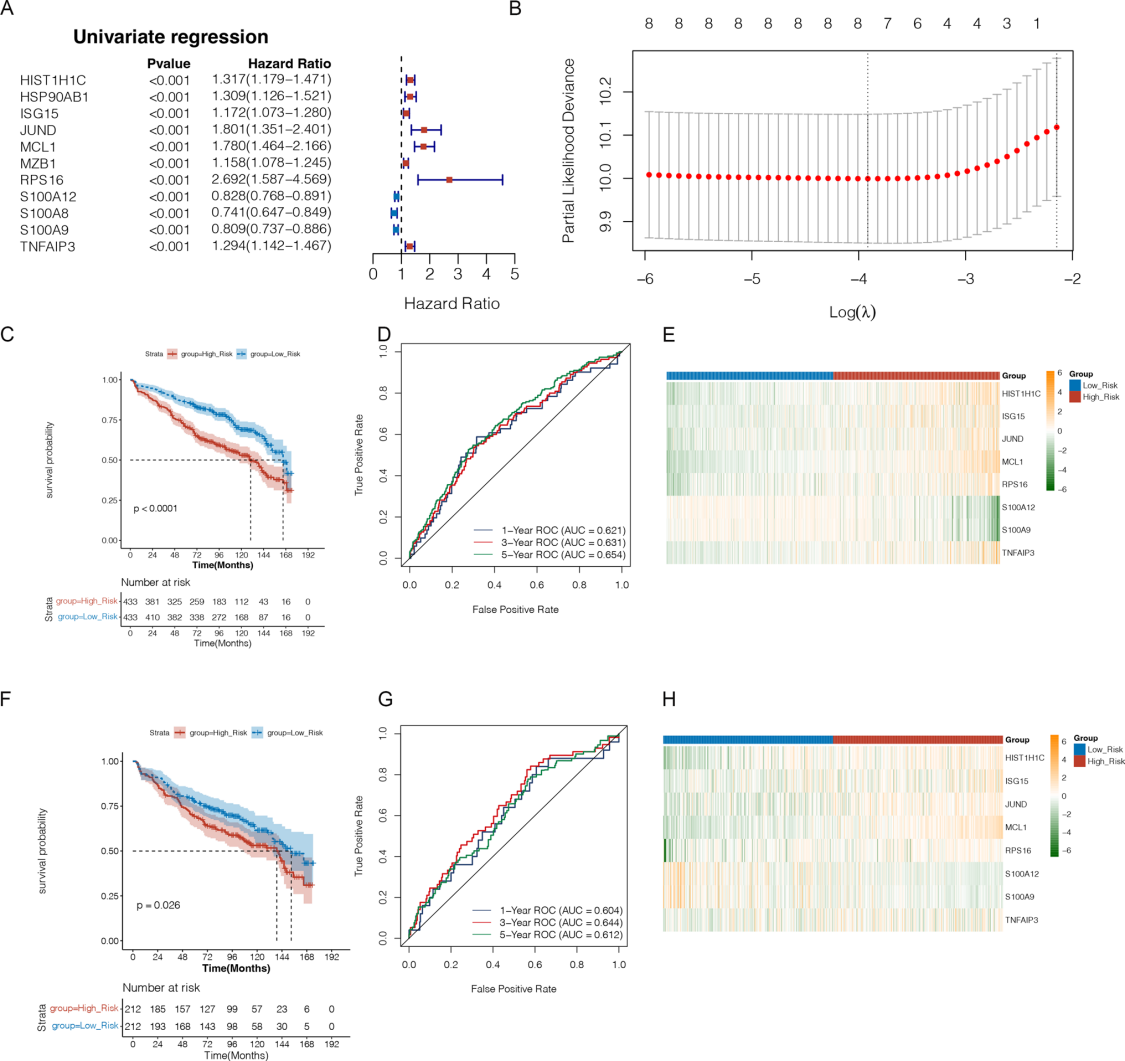
Identification and validation of potential candidate biomarkers related to DEGs-NKs. **a** Analyses of univariate Cox regression in DEGs laid out in a forest plot. **b** Selection of the optimal tuning parameter (λ) by using 20-fold cross-validation with the LASSO regression model. **c** Kaplan–Meier survival analysis of different groups based on risk score. **d** ROC curve of the risk model. **e** Heatmap of 8 hub marker genes in high and low-risk groups. **f** K-M curves for the validation set. **g** ROC curves in the validation set. **h** Heatmap of 8 hub marker genes in high and low-risk groups in the validation set.

A total of 8 biomarkers (HIST1H1C, ISG15, JUND, MCL1, RPS16, S100A12, S100A9, and TNFAIP3) were selected using univariate Cox and LASSO regression analysis (Fig. 5a–b). A risk model was constructed with the 8 biomarkers in the GSE136324 training set. Kaplan–Meier survival analysis revealed that high-risk and low-risk patients had significantly different survival outcomes (*P* < 0.0001). (Fig. 5c). The AUC values all exceeded 0.6 at 1, 3, and 5 years, which suggested that these 8 biomarkers could valuably predict survival status (Fig. 5d). The gene expression in the two risk groups was charted based on risk scores (Fig. 5e). The risk model was validated using the GSE136337 dataset(Fig. 5f–h).

### Correlation analysis of risk scores with clinical features and construction of an independent prognostic model

**Fig. 6:**
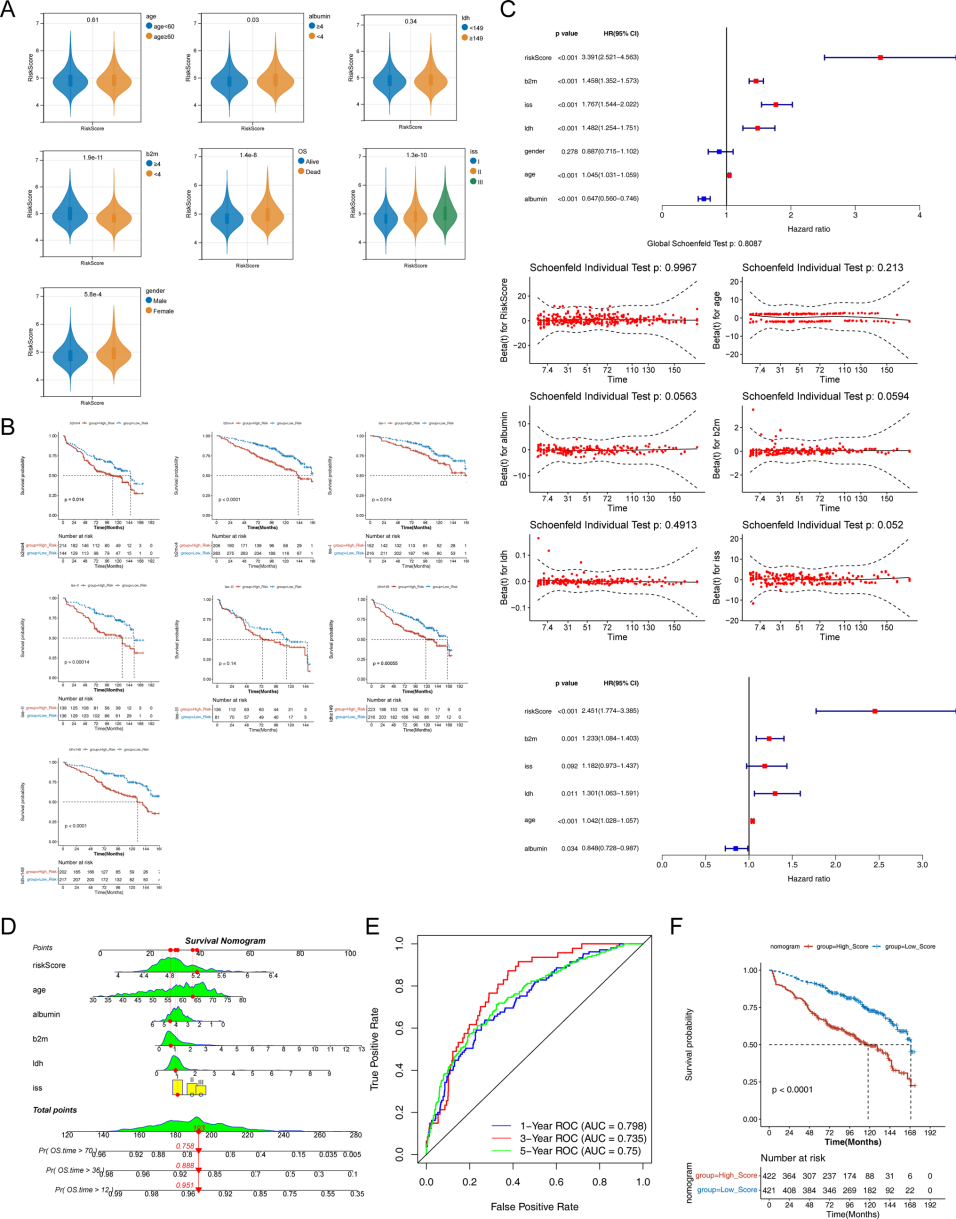
Correlation analysis of risk models and clinical characters. **a** Correlation analyses of clinical characters, and OS with risk scores. **b** A comparison of K-M survival curves for the high- and low-risk groups stratified by clinicopathologic features. **c** univariate Cox regression, PH assumption test, and multivariate Cox regression. **d** Nomogram for predicting patients’ 1, 3, and 5-year survival rates in the training set. **e** The ROC curve for the nomogram. **f** K-M survival analysis of low and high-risk groups based on nomogram

To estimate the correlation between risk scores and clinical features, we extracted the clinical information (gender, age, albumin, ß2m, LDH, ISS stage, and OS) of 866 samples from the GSE136324 dataset. Correlation analyses demonstrated marked differences existed in the risk scores in subgroups of gender, albumin, ß2m, ISS stage, and OS(Fig. 6a), and in the survival status of the samples in all 6 clinical characteristics (Fig. 6b). An assessment of age, albumin, ß2m, LDH, ISS stage, and risk scores was made using univariate Cox, PH test, and multivariate Cox regression to create a nomogram (Fig. 6c). The calibration, ROC (AUC > 0.7), and K-M curves suggested that the nomogram had favorable prediction accuracy (Fig. 6d–e).

(C) Nomogram for predicting patients’ 1, 3, and 5-year survival rates in the training set. (D) The ROC curve for the nomogram. (E) K-M survival analysis of low and high-risk groups based on nomogram.

### Landscape of immune microenvironment in two risk teams

**Fig. 7:**
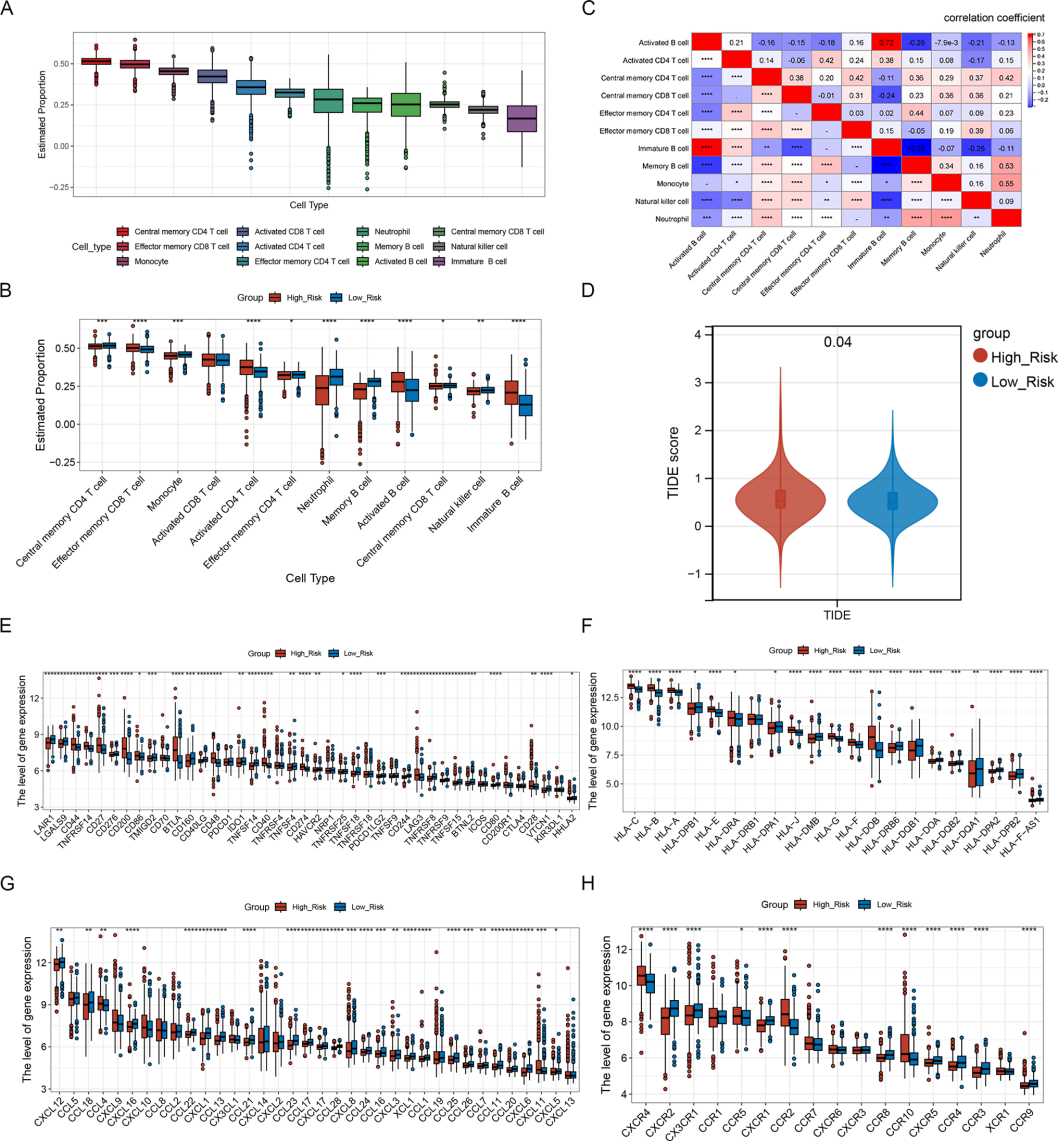
Landscape of immune microenvironment in two risk teams. **a** Infiltration of immune cells in all samples is plotted in box plots. **b** Comparative box plot of immune cell infiltration among high-risk and low-risk groups. **c** Heat map of correlations between 8 immune cell subsets. **d** Violin plot of the comparison of the TIDE scores between two groups. **e** Boxplot of expression level of 33 immune checkpoint-related genes in two risk groups. **f** Boxplot of 26 chemokines expression levels between two groups. **g** Boxplot of expression levels of 20 MHC molecules in two groups. **h** Boxplot of 12 receptor-associated gene expression levels between two groups

The abundance of immune cell infiltration was calculated for all samples using ssGSEA (Fig. 7a), and 11 immune cells differed markedly between the two risk groups: activated B cells, activated CD4 T cells, central memory CD4 T cells, central memory CD8 T cells, effector memory CD4 T cells and effector memory CD8 T cells (Fig. 7b). A correlation between 11 differentiated immune cells was also performed (Fig. 7c.) A significant difference existed in TIDE scores between the two risk groups (Fig. 7d)0.32 immune checkpoint-related genes, 20 MHC molecules, 26 chemokines, and 12 receptor-associated genes were markedly different between risk groups (Fig. 7e–h).

### Differentiation trajectories, regulatory networks, chemo drug prediction, and survival analysis for NKs

**Fig. 8:**
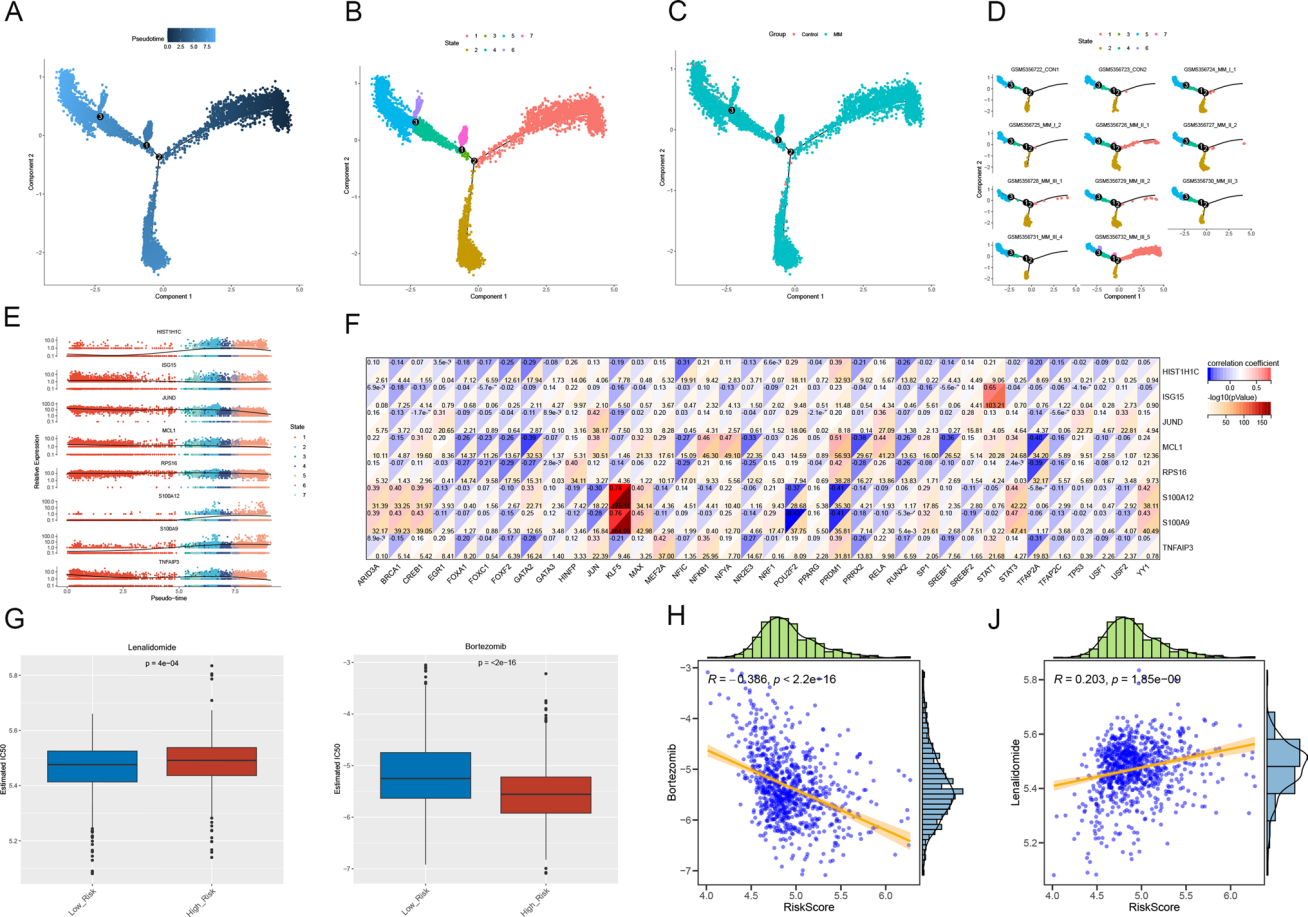
Differentiation trajectories, regulatory networks, chemo drug prediction, and survival analysis for NKs. **a**–**d**. Based on a single-cell trajectory, Monocle was able to reconstruct two main branches. Cells are colored based on state (**a**), cluster (**b**), sample (**c**), and group (**d**). (**e**) Differentially expression of biomarkers in NK cells from different branches. **f** Heatmap of hub gene and TF correlation. **g** The Comparison of IC50 of bortezomib, and lenalidomide between the high-risk and low-risk groups. **h** Scatter plots of correlation analysis of risk score and chemotherapeutic drugs

Pseudotime analysis revealed the existence of 7 distinct differentiation states of NKs (Fig. 8a–d). The expression levels of biomarkers in different differentiation states of NKs were demonstrated (Fig. 8e). Correlations between biomarkers and TF were analyzed in the training set and showed a significantly stronger negative correlation between S100A9 and POU2F2 and a significantly stronger positive correlation between S100A12 and KLF5 (Fig. 8f). There were 123 chemical drugs with notable differences in sensitivity in the inter-risk groups. Notably, bortezomib was more sensitive in the high-risk group, and lenalidomide was less sensitive in the high-risk group (Fig. 8g–j).

### Verification of diagnostic markers in clinical samples

**Fig. 9:**
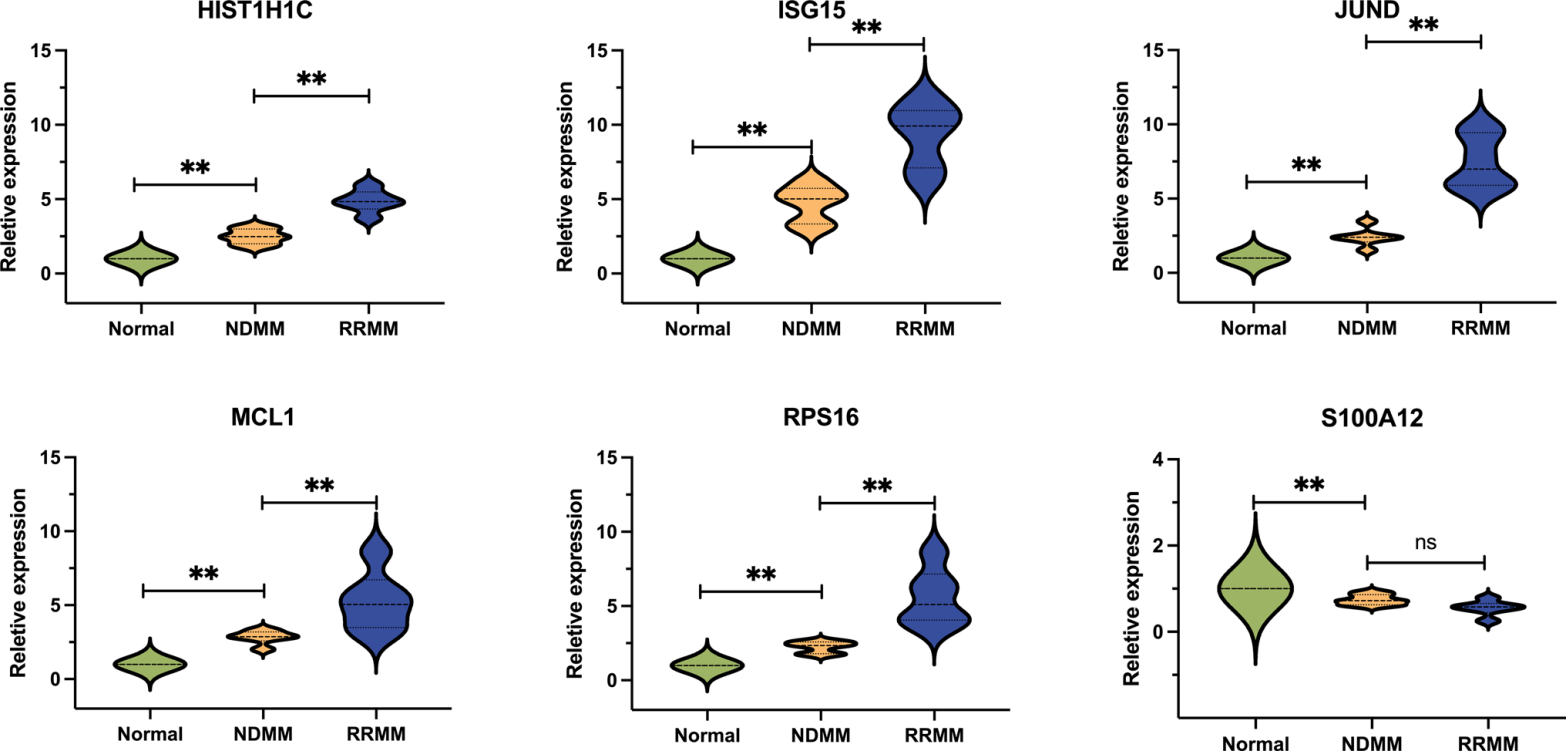
Relative expression of HIST1H1C, ISG15, JUND, MCL1, RPS16, and S100A12 in different stages of MM patients

The expression levels of eight biomarkers were assessed using qRT-PCR in a cohort of 18 bone marrow samples consisting of 6 control individuals, 6 newly diagnosed MM patients, and 6 refractory/relapsed MM patients. The results showed that MM patients exhibited elevated expression of HIST1H1C, ISG15, JUND, MCL1, and RPS16 compared to healthy controls (Fig. 9). This result suggests a potential association between the HIST1H1C, ISG15, JUND, MCL1, and RPS16 genes and disease progression and the evolution of high-risk clones.

## Discussion

Multiple myeloma is the second most prevalent hematological malignant neoplasm. Modern therapeutic approaches have significantly enhanced the overall survival rates of MM patients. However, most patients ultimately succumb to disease progression and drug resistance, which renders multiple myeloma incurable. An increasing number of studies have elucidated the pathogenesis of MM as a well-orchestrated network of immune microenvironment regulation and genomic alterations [32]. The interaction between tumor cells and the immune microenvironment plays a crucial role in fostering the spread of specific clonal populations [33]. The quantity of NK cells increases during the early stages of MM but decreases in the advanced stage of MM. This decline in NK cell function within the tumor microenvironment is attributed to the downregulation of NK cell activation receptors and the upregulation of programmed cell death receptor-1, which results in an exhausted phenotype [12, 34]. Since NK cells and MM cells are mutually immuno-edited throughout the disease via TME and anti-MM therapy. Therefore, a more comprehensive understanding of NK cells in MM patients at various disease stages would offer valuable insights for optimizing the timing and selection of immune therapies to facilitate the development of personalized medicine. In this study, we analyzed bulk and single-cell RNA sequencing datasets from MM patients to develop a transcriptional signature using NK cell marker genes to assess the extent of NK cell infiltration within the tumor microenvironment. To evaluate functional disparities in NK cells between normal and MM samples, a Hallmark pathway enrichment analysis was performed. We found the IL6-JAK-STAT3 signaling, P53 pathway, and PI3K/AKT/MTOR signaling were the most differentially expressed pathways. A previous study documented the regulation of CD38 expression in MM cells in the bone marrow microenvironment via the JAK-STAT3 pathway [35]. Interactions between MM cells and stromal cells activate the PI3K/AKT/mTOR pathway, which initiates a signaling cascade that promotes resistance to chemotherapy and cancer progression [36]. Our study found that NK cells may engage such key pathways in the disease process, which provides a potential therapeutic option in MM treatment.

To further investigate potential signaling differences within the immune microenvironment in MM, we performed cellular communication analysis. We revealed that NK cells functioned as recipients of cell–cell signaling in the major histocompatibility complex and macrophage migration inhibitory factor pathways. NK cells also exhibited robust intercellular communication with CD8 + T cells, which is consistent with findings from prior research [37].

Furthermore, a prognostic risk score model was constructed based on eight selected NK cell-related DEGs and successfully categorized patients into distinct groups. In conjunction with ISS, albumin, ß2m, LDH, and ISS stage, the risk model was identified as an independent prognostic factor. The prognostic performance of this model was subsequently validated in separate cohorts. Meanwhile, we revealed that individuals in the high-risk group demonstrated reduced levels of immune cell infiltration, particularly monocytes, B cells, and NK cells which suggests a mechanism by which these patients evade immune surveillance and hinder tumor progression. Notably, we observed that the low-risk group exhibited significantly lower TIDE scores, which suggests a reduced likelihood of tumor escape from the immune system and a heightened probability of therapeutic benefits from immune therapy.

In addition to the clinical prognostic value of the risk model, we used GDSC databases to demonstrate the practicality of identifying sensitive drugs and candidate compounds. The findings revealed a noteworthy negative correlation between the risk score and the substances Shikonin and Obatoclax. Shikonin (SHK) is a natural small agent (MW 288.3) that effectively induces apoptosis and ferroptosis in MM cells [38, 39]. The novel small-molecule antagonist obatoclax (GX015-070) demonstrated potent cytotoxic effects against MM cells derived from patients by targeting the Bcl family of proteins [40]. Also we evaluated the therapeutic effects of cornerstone therapy of MM including proteasome inhibitors and immunomodulators using this model. We found that high-score groups had a higher sensitivity to bortezomib and higher resistance to lenalidomide.

To the best of our knowledge, our results reflect pioneering efforts to exploit the genomic resources of NK cells by integrating single-cell RNA-seq and bulk RNA-seq data in the context of multiple myeloma. This integration provided novel insights into the progression of the disease and facilitated the establishment of innovative immune-related signatures based on NK cells. These signatures demonstrated their value in predicting prognosis and guiding clinicians in the selection of therapeutic strategies, particularly patients who received the triplet regimen of bortezomib, lenalidomide, and dexamethasone for first-line treatment.

However, the present study has certain limitations. Firstly, the data were derived from public databases, which could be heterogeneous, necessitating further verification of our findings through analysis of larger and more diverse datasets. Additionally, technical and equipment disparities may hinder the detection of biomarkers, potentially constraining their utilization in less-resourced or technologically underdeveloped centers. Consequently, there is a need for more prospective, randomized studies to substantiate our results. Experimental evidence is also essential to elucidate the role of NK marker genes in the progression of MM and to pinpoint precise therapeutic targets. These aspects will be investigated thoroughly in subsequent research, which we aim to detail in a forthcoming paper.

## Conclusion

The present study established an innovative NK cell-based tumorigenic and immune infiltration-associated signature that exhibited predictive ability for clinical outcomes and response to immunotherapy in MM patients. This approach holds promise for use as a prognostic biomarker, facilitating the identification of individuals who are likely to benefit from immunotherapy and aiding personalized treatment decision-making.

## Supplementary Information

Below is the link to the electronic supplementary material.

Supplementary file1 (XLS 0 kb)

Supplementary file2 (XLS 0 kb)

Supplementary file3 (XLS 20 kb)

Supplementary file4 (CSV 28 kb)

Supplementary file5 (DOCX 15 kb)
