# Supplementary material for: Exploring natural killer cell-related biomarkers in multiple myeloma: a novel nature killer cell-related model predicting prognosis and immunotherapy response using single-cell study
Source: Clin Exp Med. 2024 Apr 18;24(1):79. doi: 10.1007/s10238-024-01322-2 (PMC11026209; doi:10.1007/s10238-024-01322-2)
Supplement: Supplementary file 5 — Supplementary file5 (DOCX 15 kb) [file 10238_2024_1322_MOESM5_ESM.docx]

| HIST1H1C | Forward Primer | CCGCCTCTAAAGAGCGTAGC |
| --- | --- | --- |
|  | Reverse Primer | AGACCAAGTTTGATACGGCTG |
| ISG15 | Forward Primer | CGCAGATCACCCAGAAGATCG |
|  | Reverse Primer | TTCGTCGCATTTGTCCACCA |
| JUND | Forward Primer | TCATCATCCAGTCCAACGGG |
|  | Reverse Primer | TTCTGCTTGTGTAAATCCTCCAG |
| MCL1 | Forward Primer | TGCTTCGGAAACTGGACATCA |
|  | Reverse Primer | TAGCCACAAAGGCACCAAAAG |
| RPS16 | Forward Primer | AGGAGCGATTTGCTGGTGTAG |
|  | Reverse Primer | GAGATGGACTGACGGATAGCATA |
| TNFAIP3 | Forward Primer | TCCTCAGGCTTTGTATTTGAGC |
|  | Reverse Primer | TGTGTATCGGTGCATGGTTTTA |
| S100A12 | Forward Primer | AGCATCTGGAGGGAATTGTCA |
|  | Reverse Primer | GCAATGGCTACCAGGGATATGAA |
| S100A9 | Forward Primer | TGGAGGACCTGGACACAAATG |
|  | Reverse Primer | CACCCTCGTGCATCTTCTCG |

Primer Sequences
